# Absorption and Transport Mechanism of Red Meat-Derived *N*-glycolylneuraminic Acid and Its Damage to Intestinal Barrier Function through the NF-κB Signaling Pathway

**DOI:** 10.3390/toxins15020132

**Published:** 2023-02-06

**Authors:** Enqi He, Wei Quan, Jie Luo, Chuxin Liu, Wanting Zheng, Qingwu Shen

**Affiliations:** School of Food Science and Technology, Hunan Agricultural University, Changsha 410128, China

**Keywords:** Neu5Gc, transport mechanisms, intestinal barrier function, NF-κB, cytotoxicology

## Abstract

*N*-glycolylneuraminic acid (Neu5Gc) is a specific factor in red meat that induces intestinal disease. Our aim was to investigate the effect of Neu5Gc on the intestinal barrier as well as its mechanism of endocytosis and exocytosis. Ten specific inhibitors were used to explore the mechanism of Neu5Gc endocytosis and exocytosis by Caco-2 cells. Amiloride hydrochloride and cytochalasin D had the strongest inhibitory effect on the endocytosis of Neu5Gc. Sodium azide, dynasore, chlorpromazine hydrochloride, and nystatin also inhibited Neu5Gc endocytosis. Dynasore exhibited a stronger inhibitory effect than that of chlorpromazine hydrochloride or nystatin alone. Exocytosis inhibitors, including nocodazole, brefeldin A, monensin, and bafilomycin A, inhibited the transmembrane transport of Neu5Gc. Monensin promoted the exocytosis of Neu5Gc from Caco-2 cells. In another experiment, we observed no significant inhibitory effects of monensin and brefeldin A. Dietary concentrations of Neu5Gc induced prominent damage to intestinal tight junction proteins zonula occludens-1 (ZO-1), occludin, and claudin-1 and promoted the phosphorylation of IκB-α and P65 to activate the canonical Nuclear Factor kappa-B (NF-κB) pathway. Neu5Gc increased the RNA levels of pro-inflammatory factors IL-1β, IL-6, and TNF-α and inhibited those of anti-inflammatory factors TGF-β and IL-10. BAY, an NF-κB signaling pathway inhibitor, attenuated these changes. Reductions in the levels of ZO-1, occludin, and claudin-1 were recovered in response to BAY. Our data reveal the endocytosis and exocytosis mechanism of Neu5Gc and prove that Neu5Gc can activate the canonical NF-κB signaling pathway, regulate the transcription of inflammatory factors, thereby damaging intestinal barrier function.

## 1. Introduction

Consumption of red and processed meat is associated with intestinal diseases, especially colorectal cancer (CRC) [1]. The incidence of CRC increased from 28% to 35% and from 20% to 31% in response to the consumption of red and processed meat, respectively [2,3]. The pathogenesis of intestinal diseases caused by the consumption of red and processed meat has been ascribed to the following: (a) heterocyclic amines, polycyclic aromatic hydrocarbons, saturated fatty acids, and lipid oxidation products generated during meat processing; (b) trimethylamine oxide generated from the metabolism of choline and L-carnitine by gut microbiota; and (c) nitroso compounds (NOCs) and heme iron in processed meat products [4,5]. Several in vivo studies have confirmed the role of these substances or metabolic pathways in inducing intestinal disease; however, contradictory epidemiological data have excluded the role of intake of high fat, heterocyclic amines, polycyclic aromatic hydrocarbons, and NOCs in cancer induced by red meat and processed meat [6]. Moreover, because white meat and its processed products also contain these substances, they cannot be considered red meat-specific pathogenic factors.

*N*-glycolylneuraminic acid (Neu5Gc) is a type of sialic acid containing nine carbon atoms, which is abundant in red but not white meat and in plant foods. Most mammals, including the great apes, can spontaneously synthesize Neu5Gc. By contrast, humans cannot synthesize Neu5Gc from Neu5Ac due to a deletion mutation in the CMP-Neu5AC hydroxylase gene (*Cmah*) [7,8]. This gene is required for Neu5Gc synthesis in vivo, as shown by *Cmah*^−/−^ mouse studies. Therefore, diet is the only source of Neu5Gc for humans. Moreover, Neu5Gc can be taken up from the diet and then incorporated into cells, leading to its accumulation in the organism [9]. Part of human dietary Neu5Gc is incorporated into glycoproteins and glycolipids in multiple peripheral tissues [10]. Rombouts et al. confirmed that beef intake increased the incorporation of Neu5Gc into colon tissues of rats [11]. In vivo evidence shows that incorporated Neu5Gc can interact with circulating anti-Neu5Gc antibodies to induce immune response and chronic inflammation [12,13]. The inflammatory response triggered by a few weeks of Neu5Gc ingestion can last for years [14]. Moreover, tumor-promoted inflammation is induced when Neu5Gc interacts with anti-Neu5Gc antibodies in vivo [6,15]. Results of detailed microarray analysis show that high intake of Neu5Gc from red meat and milk results in an enhanced anti-Neu5Gc antibody level and intensive antibody–antigen reaction [16]. All these studies suggest that Neu5Gc is involved in intestinal diseases. The intestinal tract is a barrier between the internal and external environments, playing an important role in blocking toxins and food contaminants. Intestinal barrier dysfunction can lead to inflammatory bowel disease and irritable bowel syndrome [17]. Neu5Gc is absorbed in the small intestine [10]. In humans, therefore, the intestinal tract is the initial site of Neu5Gc exposure, and is also the first site to suffer the negative impacts of Neu5Gc. However, to the best of our knowledge, the action mechanism of the induction of intestinal diseases via Neu5Gc has not yet been elucidated.

Elucidating the endocytosis and exocytosis pathways of Neu5Gc through intestinal epithelial cells contributes to a better understanding of the physiological functions of Neu5Gc. Endocytosis pathways are divided into clathrin-regulated endocytosis, caveolin-regulated endocytosis, pinocytosis, and phagocytosis. Exocytosis mainly involves the endoplasmic reticulum (ER), Golgi apparatus, lysosome, vesicles, and other subcellular structures. So far, the intestinal uptake and intracellular transport of Neu5Gc has not been systematically studied.

Therefore, the first aim of this study is to systematically explore the mechanism of Neu5Gc uptake and transport by Caco-2 cells utilizing 10 endocytosis and exocytosis pathway inhibitors. Considering the significance of the intestinal barrier function for maintaining intestinal health, the second aim of this study is to reveal the mechanism of intestinal barrier dysfunction induced by Neu5Gc through investigating its effect on a Caco-2 cell monolayer model.

## 2. Results

### 2.1. Establishment and Characterization of Caco-2 Cell Monolayers

We evaluated the integrity of the Caco-2 cell monolayers by measuring its Trans Epithellal Electric Resistance (TEER, see specific protocol in Section 5.3). Meanwhile, the apparent permeability coefficient (P_app_) was also used to evaluate the integrity of Caco-2 cell monolayers (see specific protocol in Section 5.4).

**Figure 1:**
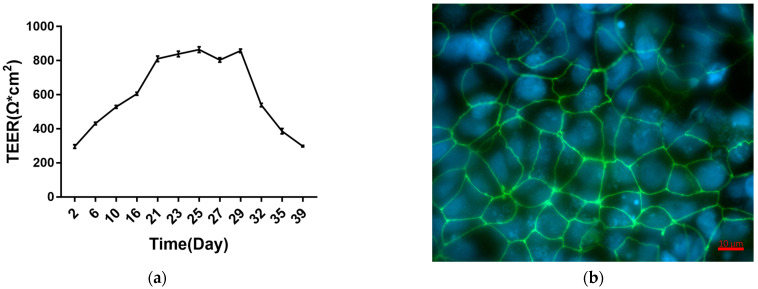
Establishment and characterization of Caco-2 cell monolayer. (**a**) Progression of Trans Epithellal Electric Resistance (TEER) of Caco-2 cells seeded on Transwell inserts. (**b**) Morphology and expression of tight junction protein zonula occludens-1 (ZO-1) in Caco-2 monolayers. The values are shown as mean ± SEM (*n* = 3).

Caco-2 cells formed monolayers in 21 d on Transwell plates. TEER measurements during this process are shown in Figure 1. Caco-2 cell monolayers entered a plateau phase on day 21 that lasted until day 29, and then TEER gradually decreased. This indicates that between days 21 and 29, Caco-2 cells formed fused monolayers. After 21 d, the apparent permeability coefficient (P_app_) and TEER values of the monolayers were measured to evaluate monolayer integrity. The results (Table 1) show that P_app_ values for Lucifer Yellow across the monolayers were less than 1 × 10^−6^ cm/s, and TEER values were greater than 800 Ω*cm^2^ after 21 d. Immunofluorescence staining (See specific protocol in Section 5.5) shows that the tight junction protein zonula occludens-1 (ZO-1) was linearly expressed after 21 d in Caco-2 cells (Figure 1b). Overall, the results indicate the establishment of Caco-2 monolayers.

### 2.2. Uptake and Transport of Neu5Gc by Caco-2 Cells

**Figure 2:**
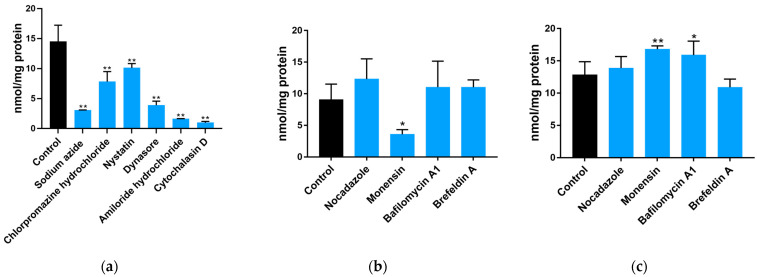
Impact of transport inhibitors on the endocytosis and exocytosis of Neu5Gc by Caco-2 cells. (**a**) Impact of inhibitors on the endocytosis of Neu5Gc was measured by detecting the amount of Neu5Gc in Caco-2 cells. (**b**,**c**) Impact of endocellular delivery inhibitors on the exocytosis of Neu5Gc was measured by detecting the content of Neu5Gc in Caco-2 cells (**b**) and the corresponding cell culture medium (**c**). The values are shown as mean ± SEM (*n* = 3), * *p* < 0.05, ** *p* < 0.01 compared with the control group.

As described in Section 5.6, the uptake and transport of Neu5Gc were explored through the effects of six and four endocytosis and exocytosis inhibitors, respectively (Figure 2a–c). The concentrations and functions of these inhibitors are shown in Table 2 [18,19,20,21]. The DMB-HPLC was conducted according to Section 5.7 to determine the content of Neu5Gc. Compared with the control group, all the endocytosis inhibitors significantly (*p* < 0.01) inhibited Neu5Gc uptake by Caco-2 cells, with amiloride hydrochloride and cytochalasin D exhibiting the strongest inhibition. Interestingly, the inhibitory effect of dynasore was stronger than that of chlorpromazine hydrochloride or nystatin alone. Nocadazole and brefeldin A had no significant effect on Neu5Gc exocytosis, whereas monensin significantly (*p* < 0.05) promoted the efflux of Neu5Gc from Caco-2 cells. Considering that the Golgi apparatus is responsible for the incorporation of Neu5Gc into glycoproteins and glycolipids, we speculate that the exocytosis of Neu5Gc from intestinal epithelial cells involves a Golgi-independent pathway.

**Figure 3:**
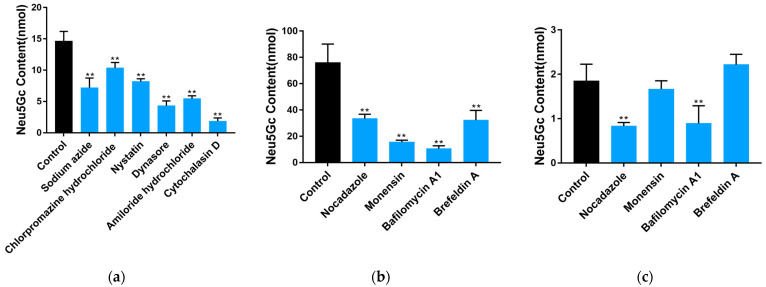
Impact of transport inhibitors on the delivery of Neu5Gc across Caco-2 cell monolayers. (**a**,**b**) Impact of endocytosis inhibitors (**a**) and endocellular delivery inhibitors (**b**) on Neu5Gc transport across Caco-2 cell monolayers. (**c**) The impact of endocellular delivery inhibitors on Neu5Gc transportation through the basolateral membrane (excluding the endocytosis of Neu5Gc and only including the exocytosis of Neu5Gc). The values are shown as mean ± SEM (*n* = 3), ** *p* < 0.01 compared with the control group.

Another experiment, as described in Section 5.8, showed that, compared with the control group, the six endocytosis inhibitors significantly (*p* < 0.01) inhibited the transmembrane transport of Neu5Gc in Caco-2 monolayers (Figure 3a), which is consistent with the results from Caco-2 cells suspension described above, while all four exocytosis inhibitors (Figure 3b) significantly (*p* < 0.01) inhibited the transmembrane transport of Neu5Gc, suggesting that tubulin, the Golgi apparatus, ER, and lysosomes are involved in the transmembrane transport of Neu5Gc. The effect of the exocytosis inhibitors on the transport of Neu5Gc to the basal lateral side of Caco-2 cell monolayers is shown in Figure 3c. Compared to the control group, we observed no significant effects of monensin and brefeldin A, indicating the presence of an exocytosis pathway for Neu5Gc that is independent of the ER and Golgi apparatus.

### 2.3. Effect of Neu5Gc on Intestinal Barrier Function

**Figure 4:**
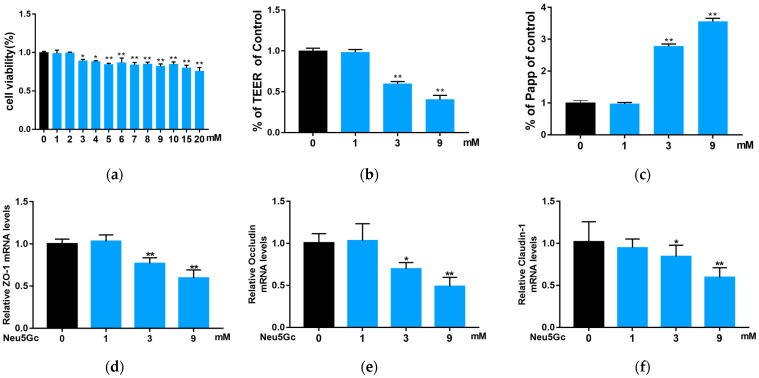

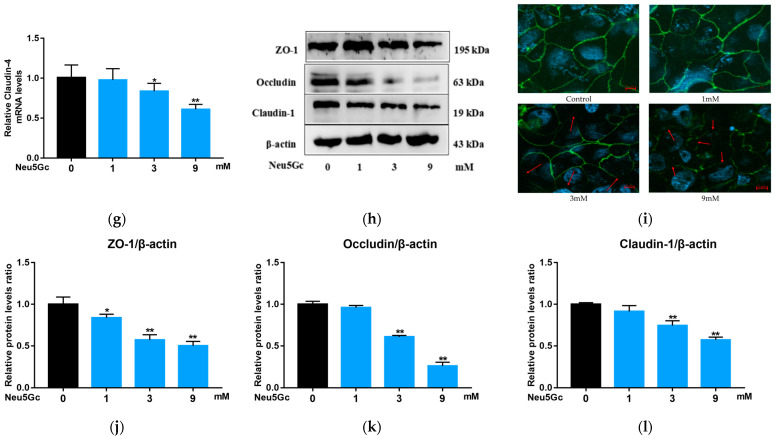
Impact of Neu5Gc on the cell viability and barrier function of Caco-2 cell monolayers. (**a**) Cell viability was detected by the MTT assay. (**b**) Impact of Neu5Gc on TEER. (**c**) Impact of Neu5Gc on P_app_, represented by Lucifer Yellow. (**d**–**g**) Impact of Neu5Gc on the mRNA expression of tight junction proteins ZO-1 (**d**), occludin (**e**), claudin-1 (**f**), and claudin-4 (**g**). (**h**) Impact of Neu5Gc on the expression of ZO-1, occludin, and claudin-1. (**i**) ZO-1 disruption induced by Neu5Gc. (**j**–**l**) Quantitative analysis of the impact of Neu5Gc on the expression of ZO-1 (**j**), occludin (**k**), and claudin-1 (**l**). The values are shown as mean ± SEM (*n* = 3), * *p* < 0.05, ** *p* < 0.01 compared with the control group.

The results of the MTT test (see specific protocol in Section 5.9) to determine the Lethal Concentration 50 (LC_50_) of Neu5Gc are shown in Figure 4a. The results show that the viability of Caco-2 cells decreased as Neu5Gc concentration increased. Concentrations from 3 to 20 mM Neu5Gc significantly (*p* < 0.05 or *p* < 0.01) decreased the viability of Caco-2 cells. Based on MTT test results, the LC_50_ of Neu5Gc is extraordinarily high, at 103.9 mM. In this study, we explored the effects of Neu5Gc on intestinal barrier function using 1, 3, and 9 mM Neu5Gc.

To evaluate whether Neu5Gc damages barrier function, Caco-2 monolayers were incubated with Neu5Gc, and we measured TEER (Figure 4b) and P_app_ for Lucifer Yellow (Figure 4c). The results show that, compared with the control group, 1 mM Neu5Gc had no significant effect on the monolayers, whereas 3 and 9 mM Neu5Gc significantly (*p* < 0.01) decreased TEER and increased P_app_ for Lucifer Yellow, indicating a prominent increase in monolayer permeability.

Typical tight junction proteins, including ZO-1, occludin, and claudin-1, are involved in intestinal barrier dysfunction [17]. To determine whether Neu5Gc has a destructive effect on intestinal barrier function, we evaluated the expression levels of ZO-1, occludin, claudin-1, and claudin-4 mRNA (see protocol in Section 5.10) after incubating cells with Neu5Gc (Figure 4d–g). Compared to the control group, 3 and 9 mM of Neu5Gc resulted in significantly (*p* < 0.05 or *p* < 0.01) lower mRNA expression levels of ZO-1, occludin, claudin-1, and claudin-4. Furthermore, Western blotting was conducted according to Section 5.11, and the results (Figure 4h,j–l) show that 3 and 9 mM Neu5Gc significantly (*p* < 0.01) reduced the expression levels of ZO-1, occludin, and claudin-1. One millimolar Neu5Gc had no significant impact on the expression levels of occludin and claudin-1, although the expression of ZO-1 was significantly (*p* < 0.05) reduced. The expression of ZO-1 was further characterized by immunofluorescence (Figure 4i), and the results indicate that the typical linear expression pattern of ZO-1 was seriously affected by Neu5Gc, i.e., ZO-1 expression became fractured and discontinuous. Overall, these results indicate that Neu5Gc reduces the expression levels of tight junction proteins and increases the permeability of Caco-2 monolayers, thus damaging the intestinal barrier function.

### 2.4. Neu5Gc Regulates the mRNA Expression of Inflammatory Factors in Intestinal Epithelial Cells through the Nuclear Factor kappa-B (NF-κB) Signaling Pathway

**Figure 5:**
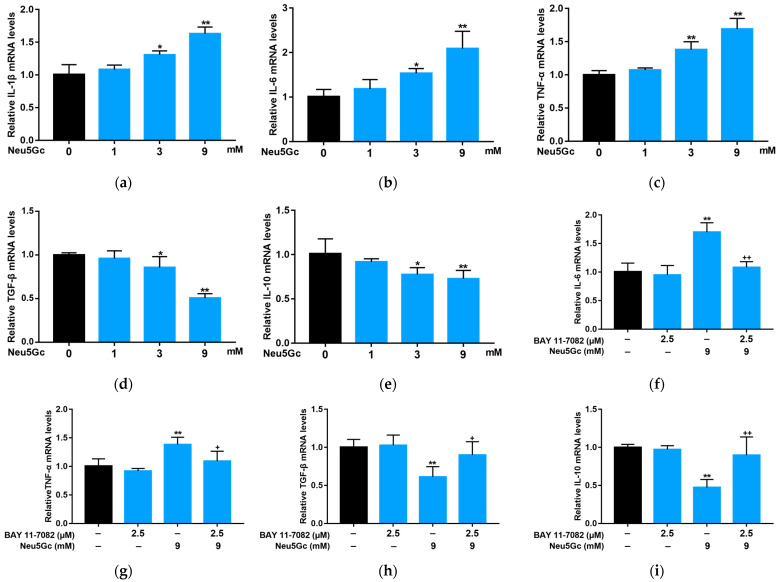
Neu5Gc regulates the mRNA expression of inflammatory factors through the nuclear factor kappa-B (NF-κB) signaling pathway. (**a**–**e**) Impact of Neu5Gc on the mRNA expression of IL-1β (**a**), IL-6 (**b**), TNF-α (**c**), TGF-β (**d**), and IL-10 (**e**). (**f**–**i**) BAY alleviates the negative effects caused by Neu5Gc on the mRNA expression of IL-6 (**f**), TNF-α (**g**), TGF-β (**h**), and IL-10 (**i**). The values are shown as mean ± SEM (*n* = 3), * *p* < 0.05, ** *p* < 0.01 compared with the control group; ^+^ *p* < 0.05, ^++^ *p* < 0.01 compared with the Neu5Gc group.

According to the method described in Section 5.10, we investigated the effects Neu5Gc on intestinal epithelial cells by measuring the mRNA expression levels of pro-inflammatory factors IL-1β, IL-6, and TNF-α and anti-inflammatory factors TGF-β and IL-10 (Figure 5). Although 1 mM Neu5Gc had no significant effect on the expression of inflammatory factors, compared with the control, 3 and 9 mM Neu5Gc significantly (*p* < 0.05 or *p* < 0.01) increased the mRNA expression levels of IL-1β (Figure 5a), IL-6 (Figure 5b), and TNF-α (Figure 5c), while significantly (*p* < 0.05 or *p* < 0.01) inhibiting the mRNA expression levels of TGF-β (Figure 5d) and IL-10 (Figure 5e). To further explore the effect of Neu5Gc on inflammatory factors, we pretreated Caco-2 monolayers with BAY, an NF-κB signaling pathway inhibitor. BAY significantly (*p* < 0.05 or *p* < 0.01) attenuated the Neu5Gc-induced changes in mRNA expression levels of IL-6 (Figure 5f), TNF-α (Figure 5g), TGF-β (Figure 5h), and IL-10 (Figure 5i). These results confirm that Neu5Gc regulates the transcription of inflammatory factors in intestinal epithelial cells through the NF-κB signaling pathway.

### 2.5. Activation of NF-κB Signaling Is Required by Neu5Gc-Induced Intestinal Epithelial Cell Barrier Dysfunction

**Figure 6:**
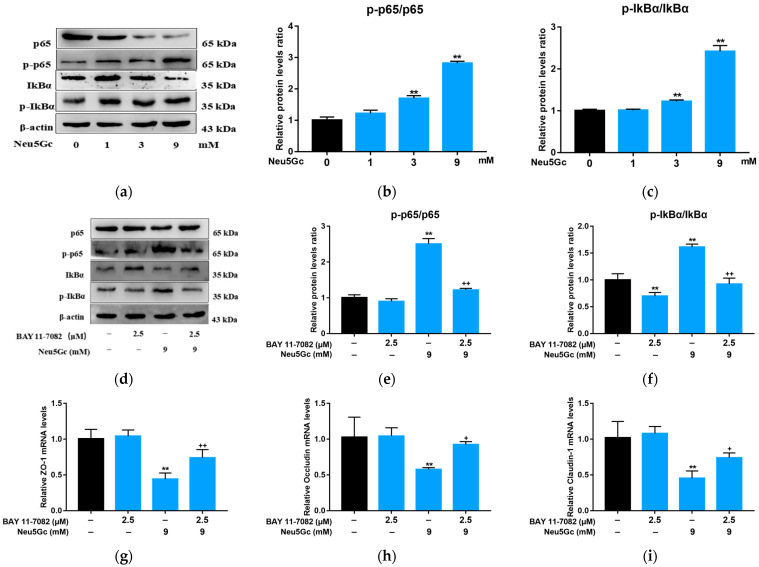

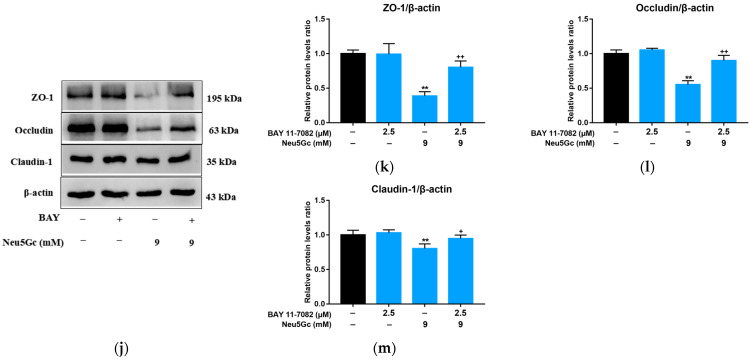
Neu5Gc induces intestinal barrier dysfunction through the activation of the NF-κB signaling pathway. (**a**–**c**) Neu5Gc facilitates the phosphorylation of p65 and IκBα (**a**), and the corresponding quantitative analysis of p-p65/p65 (**b**) and p-IκBα/IκBα (**c**). (**d**–**f**) BAY alleviates the phosphorylation of p65 and IκBα (**d**), and the corresponding quantitative analysis of p-p65/p65 (**e**) and p-IκBα/IκBα (**f**). (**g**–**i**) BAY mitigates the Neu5Gc-induced reduction of mRNA expression of ZO-1 (**g**), occludin (**h**), and claudin-1 (**i**). (**j**–**m**) Suppression of NF-κB signaling pathway contributes to the recovery of Neu5Gc-induced tight junction protein disruption (**j**), and the corresponding quantitative analysis of ZO-1 (**k**), occludin (**l**), and claudin-1 (**m**). Values are shown as mean ± SEM (*n* = 3), ** *p* < 0.01 compared with the control group; ^+^ *p* < 0.05, ^++^ *p* < 0.01 compared with the Neu5Gc group.

P65 and IκB-α play an important role in the NF-κB signaling pathway. In response to extracellular noxious stimulus, inhibitory κB kinase (IKK) is activated, which in turn dissociates and phosphorylates P65 and IκB-α. P65 subsequently enters the nucleus and induces a series of physiological reactions. Therefore, to further elucidate the mechanism of Neu5Gc-induced intestinal barrier dysfunction, the levels of phosphorylation of P65 and IκB-α were evaluated (Figure 6a). As Neu5Gc concentration increased, the phosphorylation levels of P65 (Figure 6b) and IκB-α (Figure 6c) increased. These phosphorylation levels decreased in response to BAY administration (Figure 6d–f). These results suggest that the NF-κB signaling pathway was activated by Neu5Gc. After BAY pretreatment, the monolayers were treated with 9 mM Neu5Gc, and the mRNA expression levels of ZO-1 (Figure 6g), occludin (Figure 6h), and claudin-1 (Figure 6i) were determined. The mRNA expression levels of ZO-1, occludin, and claudin-1 in the BAY + Neu5Gc group were significantly (*p* < 0.05 or *p* < 0.01) higher than those in the Neu5Gc group. Results (Figure 6j) show that Neu5Gc inhibited the expression of ZO-1 (Figure 6k), occludin (Figure 6l), and claudin-1 (Figure 6m), and their levels were significantly (*p* < 0.05 or *p* < 0.01) restored by BAY pretreatment. In conclusion, Neu5Gc activates the NF-κB signaling pathway, which induces intestinal tight junction protein destruction and ultimately leads to intestinal barrier dysfunction.

## 3. Discussion

In this study, we systematically investigated the endocytosis and exocytosis of Neu5Gc by Caco-2 cells, as well as the involvement of Neu5Gc in intestinal barrier dysfunction. Our results allow us to describe the uptake and transportation of Neu5Gc by Caco-2 cells, as well as the mechanism of Neu5Gc-induced intestinal barrier dysfunction, which involves the canonical NF-κB signaling pathway.

**Figure 7:**
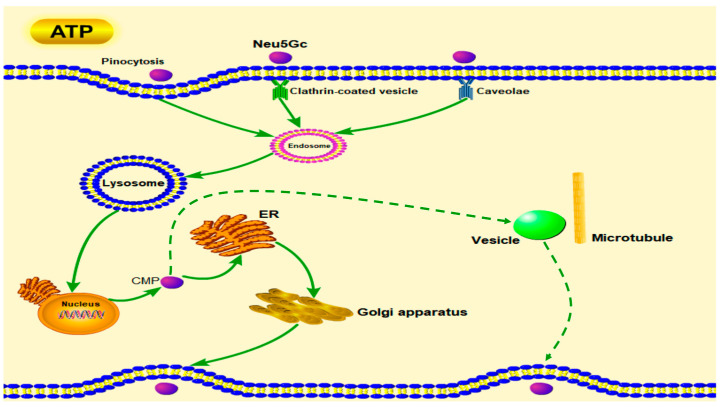
Proposed pathways for Neu5Gc uptake and transportation in human intestinal epithelial cells. Solid arrows represent certain pathways; dotted arrows represent speculated pathway.

The only study on the endocytosis and exocytosis of Neu5Gc reported that it is mainly ingested by intestinal epithelial cells through clathrin-independent pinocytosis [18]. However, the Caco-2 cell monolayers model was not used in this study, and the inhibitors tested were not enough. Therefore, the mechanisms of Neu5Gc uptake by intestinal epithelial cells and its intracellular transport were not systematically explored. In our study, the endocytosis and exocytosis of Neu5Gc were studied in detail utilizing both Caco-2 cells suspensions and monolayers. The effects of six and four endocytosis and exocytosis pathway inhibitors, respectively, were tested. The results show that sodium azide significantly (*p* < 0.01) inhibited the uptake of Neu5Gc by Caco-2 cells, suggesting that the uptake of Neu5Gc by intestinal epithelial cells is an active transport process. Amiloride hydrochloride and cytochalasin D had the strongest inhibitory effect on Neu5Gc uptake, suggesting that pinocytosis is a crucial pathway for Neu5Gc uptake. Chlorpromazine hydrochloride, and nystatin significantly (*p* < 0.01) inhibited the uptake of Neu5Gc, respectively, indicating that Neu5Gc is taken up through both clathrin- and caveolin-dependent endocytosis pathways. Moreover, the inhibitory effect of dynasore was stronger than that of chlorpromazine hydrochloride or nystatin alone, which indicates these two endocytosis pathways operate using a compensatory mechanism, i.e., when clathrin-dependent endocytosis pathway is inhibited, the caveolin-dependent endocytosis pathway remains functional, vice versa. Nocodazole, brefeldin A, monensin, and bafilomycin A1 significantly (*p* < 0.01) inhibited the transport of Neu5Gc across Caco-2 monolayers, which indicates that the transmembrane transport of Neu5Gc is complicated and involves vesicles, endoplasmic reticulum, Golgi apparatus, and lysosomes. Monensin promoted the excretion of Neu5Gc in Caco-2 cell suspensions. Meanwhile, in the exocytosis experiment conducted with Caco-2 cell monolayers, no significant effects of monensin and brefeldin A was observed. Due to the fact that the incorporation of Neu5Gc into glycoproteins and glycolipids is relevant to the ER and Golgi apparatus, we speculate that Neu5Gc has an exocytosis pathway which is independent of the ER and Golgi apparatus. A lot of endocellular vesicles can be transported directly along with microtubules through vesicle mediated motor proteins [19] and this may be the speculated exocytosis pathway mentioned above. The absorption and transport mechanism of Neu5Gc in Caco-2 cells is sketched below (Figure 7).

Neu5Gc is the only exogenous autoimmune antigen in humans [6], therefore it is of great interest to determine whether it affects intestinal barrier function and induces inflammation. Here, we show that increasing Neu5Gc concentration leads to a dose-dependent increase in P_app_. By contrast, TEER, as well as mRNA and protein expression of ZO-1, occludin, claudin-1, and claudin-4 decreased in a dose-dependent manner. ZO-1 destruction was also observed by immunofluorescence. These results indicate that Neu5Gc can destroy the integrity of the intestinal epithelium. The intestinal epithelium is the largest interface between the organism and the external environment [22]. Under normal conditions, only nutrients and water pass through the intestinal barrier [23], which greatly restricts the passage of antigens and bacteria; those that get through interact with the immune cells from the basolateral layer. However, disruption of the homeostasis of the intestinal barrier may allow more antigen and bacteria to pass, which damages mucous membranes and increases the production of reactive oxygen species. These responses lead to pathological conditions, such as inflammatory bowel disease [24].

To examine the effect of Neu5Gc on inflammatory factors and determine whether NF-κB pathway was involved, we measured the mRNA levels of various proinflammatory and anti-inflammatory factors. The results show that both 3 and 9 mM Neu5Gc can increase the mRNA expression levels of pro-inflammatory factors and decrease the levels of anti-inflammatory factors through the NF-κB pathway. Neu5Gc causes lots of clinical consequences. In xenotransplantation studies, Neu5Gc, which binds α-galactose at the end of the sugar chain, was found to be the major exogenous antigen that triggers inflammation and leads to tissue rejection [25,26,27]. Study showed that the overexpression of Neu5Gc in the nervous system caused a variety of harms such as impaired motor activity and dysmnesia [28]. Neu5Gc-induced chronic inflammation may be the red meat-specific pathogenic mechanism of atherosclerosis, hepatocellular carcinoma, type 2 diabetes mellitus, and other red meat-related diseases [4,12]. It is worth noting that all these clinical consequences are related to the inflammation induced by the interaction between Neu5Gc and Neu5Gc-antibody. Normally, humans have a rich and diverse antibody spectrum against Neu5Gc-glycans, along with the production of anti-Neu5Gc antibodies with different titers [29,30,31]. Interaction of Neu5Gc with human anti-Neu5Gc antibodies induces chronic inflammation [12,13], which may contribute to the development and progression of diet-related cancers, such as CRC [13,32,33,34,35]. The combination of anti-Neu5Gc antibodies and Neu5Gc-expressing tumor can further promote tumor growth [29], resulting in a vicious cycle. Several in vivo studies have demonstrated that Neu5Gc increases the risk of cancer by interacting with anti-Neu5Gc antibodies to induce “xenosialicacid inflammation” [12,36,37]. Neu5Gc can also act as a nitrogen and carbon source for *Escherichia coli* K-12 and promote its proliferation, which produces LPS and induces intestinal inflammation. This process is regulated by NF-κB and LC3B pathways [38].

The results of this study indicate that Neu5Gc promotes the phosphorylation of IκB-α and P65, which activates the canonical NF-κB pathway. BAY, an NF-κB signaling pathway inhibitor mitigated this change while restoring the Neu5Gc-induced decrease in the expression of intestinal ZO-1, occludin, and claudin-1. Overall, these results suggest that Neu5Gc can activate the canonical NF-κB signaling pathway and promote the phosphorylation of IκB-α and p65, which in turn induces the entry of p65 into the nucleus, resulting in a series of biochemical reactions that culminate in the destruction of tight junction proteins and intestinal barrier damage. This process likely involves the activation of the Myosin Light-Chain Kinase (MLCK) signaling pathway [39,40]. Similar studies have shown that IL-1β, mono-carboxylate transporter 4, and ochratoxin A regulate intestinal cell barrier function through the NF-κB signaling pathway [40,41,42], which operates through a mechanism previously described [43]. Using specific inhibitors and siRNA technology, Rana Al-Sadi et al. demonstrated that IL-1β increases tight junction permeability of intestinal epithelial cells through the MEKK—IKK—NF-κB—MLCK pathway [40,44]. Disruption of the intestinal barrier by TNF-α is also modulated by the canonical NF-κB pathway [39]. Considering the pro-inflammatory effect of Neu5Gc (Figure 5), the vicious cycle involving these two pro-inflammatory factors (IL-1β and TNF-α) in the NF-κB signaling pathway may further amplify the destruction of intestinal barrier function induced by Neu5Gc.

## 4. Conclusions

In this study, we systematically explored the mechanisms of endocytosis and exocytosis of Neu5Gc in intestinal epithelial cells. The endocytosis of Neu5Gc is an active transport process. Pinocytosis playes an very important role in the endocytosis of Neu5Gc. Both clathrin- and caveolin-dependent endocytosis pathways are involved in Neu5Gc endocytosis. The endoplasmic reticulum, Golgi apparatus, lysosome, and vesicles participate in the intracellular transportation of Neu5Gc. In addition, there is a possibility that the Neu5Gc exocytosis pathway in intestinal epithelial cells is independent of the Golgi apparatus; however, the specific mechanism remains unclear. The results of this study demonstrate that Neu5Gc can regulate the expression levels of mRNAs for inflammatory factors in intestinal epithelial cells, and it can destroy intestinal barrier function by activating the canonical NF-κB signaling pathway. Thus, our study provides a novel insight into the absorption and transportation of Neu5Gc in intestinal epithelial cells as well as its biohazard to intestinal health.

## 5. Materials and Methods

### 5.1. Chemicals and Reagents

Neu5Gc was purchased from Aladdin (Shanghai, China). Dulbecco’s modified Eagle’s medium (DMEM), trypsin, fetal bovine serum, penicillin, streptomycin, phosphate-buffered saline (PBS), Hank’s Balanced Salt Solution (HBSS) were purchased from BI (Kibbutz Beit-Haemek, Israel). Mouse anti-ZO-1 antibody Alexa Flour 488 conjugate were purchased from Invitrogen (San Francisco, CA, USA). Anti-ZO-1 and occludin antibodies were purchased from Affinity (Changzhou, China). Anti-claudin-1, p65, p-p65, I-κBα, p-I-κBα, and anti-β-actin antibodies were purchased form Abmart (Shanghai, China). BAY 11-7082 was purchased from Beyotime (Shanghai, China). Fish skin gelatin, 1,2-diamino-4,5-methylene-dioxybenzene (DMB), and sodium azide was purchased from Sigma (St. Louis, MI, USA). Chlorpromazine hydrochloride, amiloride hydrochloride and the MTT Detection Kit were purchased from Solarbio (Beijing, China). Dynasore was purchased from MCE (Monmouth Junction, NJ, USA). Nocodazole and nystatin were purchased from Yuanye Bio-Tech (Shanghai, China). Monensin sodium salt and cytochalasin D were purchased from Aladdin (Shanghai, China). Bafilomycin A1 was purchased from LC Laboratories (Woburn, MA, USA). Brefeldin A was purchased from Selleckchem (Houston, TX, USA).

### 5.2. Cell Culture and Neu5Gc Treatment

Caco-2 intestinal epithelial cells were purchased from the ATCC (Rockville, MD, USA) and cultivated at 37 °C and 5% CO_2_ in DMEM medium supplemented with 10% FBS and 1% antibiotics (penicillin/streptomycin). Caco-2 cells were seeded in 6-well plates, 96-well plates, or Transwell polyester membrane inserts and cultivated in a serum-free medium (BIO-MPM-1; BI, Israel) prior to each experiment to eliminate the impact of Neu5Gc in the FBS. The Neu5Gc was dissolved in serum-free culture medium or HBSS solution for the consequent experiment. The toxicity of Neu5Gc was evaluated through the MTT test (described in Section 5.9), and based on the results, a nontoxic concentration of 0.6 mM Neu5Gc was used to examine the mechanism of endocytosis and exocytosis (described in Section 5.6 and Section 5.8). The quantification of Neu5Gc in the Caco-2 cells was conducted by DMB-HPLC (described in Section 5.7). Because ~85% of people consume between 2.92 and 8.76 mmol Neu5Gc per year [16], and the LC_50_ value of Neu5Gc (103.9 mM) is extremely high (described in Section 2.3), we choosed 1, 3, and 9 mM Neu5Gc for exploring the Neu5Gc induced damage to intestinal barrier function. Specifically, Caco-2 cells were treated with a solution containing 1, 3, and 9 mM Neu5Gc for 24 h. Then, the TEER (described in Section 5.3) and Papp (described in Section 5.4) of Caco-2 cell monolayers were measured. Subsequently, the inflammatory cytokines and tight-junction proteins mRNA expression (described in Section 5.10), tight-junction proteins and NF-κB related proteins expression (described in Section 5.5 and Section 5.11) were detected. In another experiment, Caco-2 cells were pretreated with BAY, a specific inhibitor of NF-κB signaling pathway, for 2 h, and then incubated with 9 mM Neu5Gc for 24 h, and the related indicators mentioned above were determined.

### 5.3. TEER Analysis

Trans Epithellal Electric Resistance (TEER) is a common indicator to measure the integrity of cell monolayers. TEER is positively correlated with cell monolayers integrity and usually measured by TEER detector. In this study, Caco-2 cells were seeded on Transwell polyester membrane inserts (12 wells, 0.4 μm pores, Corning, NY, USA) and grown until they formed confluent monolayers. TEER was measured utilizing the Millicell-Electrical Resistance System (Merck KGaA, Darmstadt, Germany). TEER values are expressed as Ω*cm^2^ [42].

### 5.4. Measurement of P_app_

The apparent permeability coefficient (P_app_) is another common indicator to measure the integrity of cell monolayers. The P_app_ is negatively correlated with cell monolayers integrity and is characterized by measuring the amount of certain hydrophilic molecules passing through the cell monolayers. In this study, P_app_ assays used Lucifer-Yellow (Sigma, St. Louis, MI, USA) applied to the apical side of the Caco-2 cell monolayers. After 1 h incubation with Lucifer-Yellow, samples were collected from the basolateral side of the monolayers, and their fluorescence was measured using fluorescence spectrophotometer (Hitachi, Japan). P_app_ was calculated according to the equation below [45].

P_app_ = (dQ/dt)(1/AC_0_)

dQ/dt is the steady-state flux (μmol/s), A is the surface area of the filter (cm^2^) and C_0_ is the initial concentration in the donor chamber (μM).

### 5.5. Immunofluorescence

Caco-2 cells (treated with or without Neu5Gc) were fixed with 4% paraformaldehyde for 15 min at room temperature and then blocked with 1% fish gelatin (Sigma, St. Louis, MI, USA) for 1 h. Cells were then incubated with a mouse anti-ZO-1 antibody-Alexa Flour 488 conjugate at a concentration of 5 μg/mL for 1 h in the dark. After nuclei were stained with DAPI (Southern biotech, Birmingham, AL, USA), the images were captured using laser scanning confocal microscopy (Carl Zeiss, Oberkochen, BW, Germany).

### 5.6. Endocytosis and Exocytosis Pathways of Neu5Gc in Caco-2 Cells

We explored the endocytosis and exocytosis pathways by pretreating Caco-2 cells with different inhibitors (Table 2) as reported [19,46].

We investigated endocytosis pathways by first pretreating Caco-2 cells with endocytosis inhibitors at 37 °C for 2 h. The cells were then incubated with 0.6 mM Neu5Gc containing the same inhibitors for 12 h. Subsequently, the cells were collected for Neu5Gc quantification by DMB-HPLC. The amount of Neu5Gc is expressed relative to the total proteins detected by the BCA protein assay kit (Beyotime, Shanghai, China).

We investigated exocytosis pathways by first incubating Caco-2 cells with 0.6 mM Neu5Gc at 37 °C for 8 h. Thereafter, the culture medium was aspirated, and the cells were washed three times with PBS to remove Neu5Gc. Subsequently, the cells were treated with exocytosis inhibitors for 6 h. Finally, the cells and culture medium were separately collected for Neu5Gc quantification by DMB-HPLC. The results are expressed relative to total proteins detected by the BCA assay.

### 5.7. Quantification of Neu5Gc by DMB-HPLC

Caco-2 cells and the culture medium were collected, respectively. The Neu5Gc from these samples were tagged with DMB (a fluorogenic substrate) as described [12,47] and quantified by HPLC using a C18 column (Waters, Milford, MA, USA). HPLC was performed using a mobile phase consisting of 88% H_2_O, 7% methanol, and 5% MeCN at a flow rate 0.9 mL/min. Fluorescent signals were measured at excitation and detection wavelengths of 373 and 448 nm, respectively.

### 5.8. Endocytosis and Exocytosis Pathways of Neu5Gc in Caco-2 Cell Monolayers

Caco-2 cell monolayers growing on Transwell polyester membrane inserts were pretreated with transport inhibitors (Table 1) at 37 °C 2 h. Then, the monolayers were incubated with 0.6 mM Neu5Gc containing the corresponding inhibitors for 12 h. Subsequently, the amount of Neu5Gc transported to the basolateral side was measured by DMB-HPLC.

In a separate experiment, the Caco-2 cell monolayers were pretreated with 0.6 mM Neu5Gc at 37 °C for 8 h. Subsequently, the Caco-2 cell monolayers were rinsed three times with HBSS to eliminate remove Neu5Gc, followed by treatment with endocellular transport inhibitors (Table 1) for 6 h. Thereafter, the amount of Neu5Gc transported to the basolateral side was measured by DMB-HPLC.

### 5.9. Cell Viability Analysis

Logarithmic growth phase Caco-2 cells were seeded in 96-well plates at an inoculum density of 5 × 10^4^ cells/mL. Different concentrations of Neu5Gc (1 mM to 20 mM) were added and the mixture was incubated for 24 h. Thereafter, MTT and formazan solutions were added according to the MTT Cytotoxicity Assay Kit instructions (Solarbio, Beijing, China). Cell viability was calculated based on absorbance at 490 nm (Thermo Fisher, Waltham, MA, USA).

### 5.10. Quantitative Real-Time PCR

Total RNA from Caco-2 cells was extracted using a Trizol reagent (TaKaRa, Beijing, China) and then transcribed into complementary DNA using the Prime Script RT reagent kit (Takara, Beijing, China) according to the manufacturer’s protocol. RNA quality was determined based on the ratio of OD260/OD280. Real-time PCR was performed with the Step One Plus detection system (Applied Biosystems, Waltham, MA, USA) using the SYBR Premix Ex Taq II kit (TaKaRa, Beijing, China) according to the manufacturers’ protocols. The PCR procedure comprised the following steps: 95 °C step for 30 s, followed by 40 cycles at 95 °C for 5 s and 60 °C for 30 s. The melting curve showed only one peak for each PCR product. The 2^−ΔΔCt^ was calculated to represent relative mRNA levels of target genes, with GAPDH as a housekeeping gene for normalization. The primers were synthesized by Sangon Biotech (Shanghai, China). Primer sequences are shown in Table 3.

### 5.11. Western Blot Analysis

Caco-2 cells were lysed with cold RIPA buffer (Beyotime, Shanghai, China) supplemented with the protease inhibitor PMSF (Beyotime, Shanghai, China). Total protein was quantified using the BCA protein assay kit (Beyotime, Shanghai, China). Total proteins (40 ± 10 µg) were loaded on 12% SDS-PAGE gels and transferred to polyvinylidene fluoride membranes (Sigma Millipore, Billerica, MA, USA). The membranes were blocked for 2 h and then incubated with primary antibodies overnight at 4 °C, followed by incubation with secondary antibodies for 1 h. The density of each protein was assessed by the ECL detection kit (Beyotime, Shanghai, China).

### 5.12. Statistical Analysis

SPSS software version 26.0 was used for the statistical analysis. The significance of differences between mean values was determined with analysis of variance (ANOVA), followed by Tukey’s least significant difference (LSD) test. The results are expressed as mean ± standard error of mean (SEM). Differences were considered as statistically significant at *p* < 0.05.

## Figures and Tables

**Table 1:** Apparent permeability coefficient (P_app_) and TEER of Caco-2 monolayers.

Indicators	Monolayer 1	Monolayer 2	Monolayer 3	Monolayer 4	Monolayer 5	Monolayer 6	Monolayer 7
P_app_ (×10^−6^ cm/s)	0.0210 ± 0.0014	0.0322 ± 0.0039	0.0305 ± 0.0029	0.0283 ± 0.0025	0.0268 ± 0.0025	0.0286 ± 0.0028	0.0257 ± 0.0027
TEER (Ω*cm^2^)	974.40 ± 20.08	859.79 ± 44.82	808.27 ± 39.46	949.01 ± 30.79	936.69 ± 40.51	961.71 ± 29.04	1022.56 ± 17.51

The values are shown as mean ± SEM (*n* = 3).

**Table 2:** Concentrations and functions of endocytosis and exocytosis inhibitors.

Inhibitor	Classification	Concentration	Function
Sodium azide	Endocytosis	1 mg/mL	Inhibit energy-dependent active transportation
Chlorpromazine	Endocytosis	30 μM	Inhibit clathrin-dependent endocytosis
Nystatin	Endocytosis	25 μg/mL	Inhibit caveolin-dependent endocytosis
Amiloride hydrochloride	Endocytosis	3 mM	Inhibit macropinocytosis and phagocytosis
Dynasore	Endocytosis	80 μM	Inhibit both clathrin- and caveolin-dependent endocytosis
Cytochalasin D	Endocytosis	10 μM	Inhibit macropinocytosis and phagocytosis
Nocodazole	Exocytosis	6 μg/mL	Inhibit microtubule-vesicle mediated protein transport to membranes
Monensin	Exocytosis	Monensin	Inhibit the transportation between Golgi apparatus and cell membranes
Bafilomycin A1	Exocytosis	100 nM	Inhibit the maturation process of early endosomes to lysosomes
Brefeldin A	Exocytosis	25 μg/mL	Inhibit the transportation between endoplasmic reticulum (ER) and Golgi apparatus

**Table 3:** Parameters of primers for inflammatory cytokines, tight junction proteins, and GAPDH.

Genes	Accession Number	Primers	Sequences	Product Length (bp)
ZO-1	NM_001301025.3	Forward Reverse	CAGAAATACCTGACGGTGCT TCCATTGCTGTGCTAGTGAG	233
Occludin	NM_021101.4	Forward Reverse	CCGGCGACAACATCGTGAC CGGGTTGCTTGCAATGTGC	136
Claudin-1	NM_002538.2	Forward Reverse	AAGAGTTGACAGTCCCATGGCATAC ATCCACAGGCGAAGTTAATGGAAG	133
Claudin-4	NM_001305.5	Forward Reverse	AGACTTCTACAATCCGCTGG ACCTTACACGTAGTTGCTGG	199
IL-Iβ	NM_000576.3	Forward Reverse	AGGCACAAGGCACAACAGGCT GTCCTGGAAGGAGCACTTCATCTGT	187
IL-6	NM_001371096.1	Forward Reverse	AGACAGCCACTCACCTCTTCAG TTCTGCCAGTGCCTCTTTGCTG	132
TNF-α	NM_000594.4	Forward Reverse	CTCTTCTGCCTGCTGCACTTTG ATGGGCTACAGGCTTGTCACTC	135
TGF-β	NM_000660.7	Forward Reverse	AACCCACAACGAAATCTATGAC GCTGAGGTATCGCCAGGAAT	205
IL-10	NM_000572.3	Forward Reverse	GCTCTTGCAAAACCAAACCAC TGGCAACCCAGGTAACCCTTA	286
GAPDH	NM_001357943.2	Forward Reverse	GTCTCCTCTGACTTCAACAGCG ACCACCCTGTTGCTGTAGCCAA	131

## Data Availability

The data presented in this study are available on request from the corresponding author. The data are not publicly available due to privacy restriction.
